# Supplementary material for: Prevalence of text neck posture, smartphone addiction, and its association with neck disorders among university students in the Kingdom of Saudi Arabia during the COVID-19 pandemic
Source: PeerJ. 2022 Dec 15;10:e14443. doi: 10.7717/peerj.14443 (PMC9760021; doi:10.7717/peerj.14443)
Supplement: Supplemental Information 2 [file peerj-10-14443-s002.pdf]

Raw Data – Code Book

1. S.No – Serial number allotted to each participant
2. Age – in years
3. Gender – (1= Male, 2 = Female)
4. Height – in Centimetres (cm)
5. Weight – in Kilograms (kg)
6. BMI – Body mass index in kg/m^2
7. Han-Dom- Hand dominance (1 = Left and 2 = Right)
8. Department – 1- Physical therapy, 2- Nursing, 3- Medical Laboratory, 4- Medical Equipment Technology, 5-Public Health, 6- Medical Imaging
9. Educational Qualification – 1- Bachelor, 2- Master, 3- PhD
10. Ph- years- Smartphone usage in years, Tab- years- Tab usage in years, Lap- years- Laptop usage in years
11. Ph- Daily- Average hours of Smartphone usage per day (1=Less than 1 hour, 2=About an hour, 3= 1-3 hours, 4=3-5 hours, 5= 5-7 hours, 6=7 hours or more)
12. Duration of Smartphone usage, Ph -Study = For study purpose, Ph- Soc= For social media, Ph- Game = For games (1=Less than 1 hour, 2=1-2 hours, 3= 2-3 hours, 4=3-4hours, 5= 4 hours or more)
13. Ph- Hold = Manner of holding a smartphone (1- Right hand, 2- Left hand, 3- Both hands, 4- Use cradle, stand or any other accessories)
14. Text neck- Presence of Text neck posture (1- A, 2- B, 3- C, 4-D corresponding to postures illustrated in Figure 1)
15. Neck MS- Presence of neck disorder- (0- Absent, 1- Present)
16. SAS- Tot- Smartphone addiction scale total score
17. Category based on Smart phone addiction score (1- No overuse or Addiction, 2- Overuse or addiction)
18. IP MET- METs calculated based on International Physical Activity Questionnaire
19. IP-CAT- Category of Physical activity based on International Physical Activity Questionnaire (1- Light, 2- Moderate, 3-Vigorous)
